# Exon skipping caused by a complex structural variation in *SH2D1A* resulted in X‐linked lymphoproliferative syndrome type 1

**DOI:** 10.1002/mgg3.1873

**Published:** 2022-01-29

**Authors:** Liwen Wu, Feng Yang, Jia Wang, Fan Yang, Mengmeng Liang, Haiyan Yang

**Affiliations:** ^1^ Department of Neurology Hunan Children 's Hospital Changsha P.R. China; ^2^ Cipher Gene LLC Beijing China

**Keywords:** case report, genomic structural variants, *SH2D1A*, whole exome sequencing, XLP1

## Abstract

**Background:**

X‐linked lymphoproliferative syndrome type 1 (XLP1) is a rare primary immunodeficiency disorder characterized by severe immune dysregulation often after viral infection. It is caused by hemizygous mutations in the X‐linked *SH2D1A* gene. People with XLP1 have complex and variable phenotype manifestations as EBV‐driven severe or fulminant mononucleosis, hemophagocytic lymphohistiocytosis (EBV‐HLH), dysgammaglobulinemia, and B‐cell lymphoma.

**Methods:**

Immunological analyses, clinical laboratory testing, and whole exome sequencing (WES) were performed to help the disease diagnosis for the patient with severe immune dysregulation. Routine and extended WES analysis pipelines were applied to explore candidates. A complex genomic structural variation in *SH2D1A* was detected and verified by Inverse‐PCR, Gap‐PCR, and RT‐PCR.

**Results:**

Here we reported that a five‐year‐old male patient manifested with EBV‐HLH, recurrent infection by severe immune dysregulation, and successfully managed with HSCT. He finally established precise disease diagnosis as XLP1 caused by a complex genomic structural variation in *SH2D1A* (NC_000023.11:g. [124,350,560_124365777del; 124,365,777_124365917inv; 124,365,911_124365916del]). The mother and grandmother of the proband were confirmed to be carriers. The complex variant resulted in the exon 2 skipping and was predicted to generate a prematurely truncated protein.

**Conclusion:**

The complex structural variant combined with paracentric inversion and large size deletions was first reported in XLP1 cases. It is considered to be pathogenic based on the truncation of the mRNA sequence and cosegregation with the disease in three‐generation pedigree analysis. This finding has expanded the known XLP‐related mutation spectrum in Chinese patients and indicated remarkable effects on the early diagnosis and therapeutic implication using proper molecular testing techniques.

## Graphical abstract

Here we reported a five‐year‐old male patient with recurrent infection by severe immune dysregulation, he finally established precise disease diagnosis as XLP1 caused by a complex genomic structural variation in *SH2D1A*. Our finding has expanded the known XLP‐related mutation spectrum in Chinese patients and indicated remarkable effects on the early diagnosis and therapeutic implication using proper molecular testing techniques.

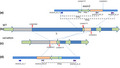

## 1 INTRODUCTION

X‐linked lymphoproliferative EBV‐HLH type 1 (XLP1; OMIM 308240) is a rare primary immunodeficiency affecting approximately 1–2 per 1 million males, which was first described in 1970s (Purtilo et al., 1974). It is an X‐linked recessive genetic disorder caused by mutations in *SH2D1A* gene, which encodes the intracellular adaptor molecule, referred to as SAP for SLAM‐associated protein. SAP is mainly expressed in T cells and NK cells, it regulates the signal transduction pathways downstream of the SLAM family of surface receptors to control the function of CD4+ T cell (and by extension B cells), CD8+ T cell, and NK cells, as well as the development of NKT cells (Tangye, 2014), and the deficiency may lead to the cellular and humoral immune abnormity characterized in patients.

XLP1 is characterized by severe immune dysregulation often after viral infection, typically with Epstein–Barr virus (EBV). It has complex phenotype manifestations such as severe or fatal mononucleosis, acquired hypogammaglobulinemia, hemophagocytic lymphohistiocytosis (HLH), and/or malignant lymphoma. Other features may include unremitting fever, aplastic anemia, red cell aplasia, splenomegaly, cytopenia, and lymphomatoid granulomatosis (Booth et al., 2011). It has a strong resemblance to that of HLH (Arico et al., 2001) which is a rare, complex, life‐threatening hyper‐inflammatory disease due to the excessive activation of lymphocytes mediated secretory cytokines in the body (Sheth et al., 2019). Many pediatric patients usually suffer from an expanding spectrum of genetic diseases that can be complicated by the syndrome of HLH.

Here we reported a five‐year‐old male patient who was finally diagnosed as XLP1 based on the novel complex genomic structural variants: NC_000023.11:g.[124,350,560_124365777del; 124,365,777_124365917inv; 124,365,911_124365916del], referred to NM_002351.4: [c.154_201+87inv; IVS1del15kb] in *SH2D1A* by genomic breakpoint detection using extended whole exome sequencing (WES) and amplicon sequencing. The structural variation affected 5 prime‐ splice acceptor site, branch point, and other splicing regulator regions of exon 2. It caused exon 2 skipping and was predicted to induce a frameshift leading to premature termination of SAP. Furthermore, a three‐generation pedigree analysis validated cosegregation with disease and X‐linked recessive inheritance pattern of XLP1 in the family.

## 2 METHODS

### 2.1 Patient

Informed consent was obtained from the parents and their families. This study was approved by the institutional review board of the Hunan Children's Hospital. Clinical and laboratory data were collected from the patients' medical records, including clinical manifestations, laboratory tests, treatments, and outcomes. Blood from the patient and his family were collected and transferred to our laboratory for analysis within 24 h of collection.

### 2.2 Whole exome sequencing (WES)

Samples from the patient and his family members were prepared as follows: 2 ml of whole blood from the patient and his parents were respectively drawn into EDTA‐Vacutainer tubes. Genomic DNA was extracted from the peripheral blood mononuclear cells (PBMCs) by using QIAamp DNA Mini Kit (QIAGEN) and fragmented by Covaris S2 sonicator. DNA concentrations were measured by Qubit 3.0 fluorometer (Invitrogen).

Fragmented DNA was used for library preparation using NadPrep DNA Universal Library Preparation Kit (Nanodigmbio) according to the manufacturer's protocol. The quality and concentration of the libraries were verified using the Agilent 2100 Bioanalyzer and Qubit3.0 Fluorometer, respectively. Target enrichment of all libraries was conducted by xGen Exome Research Panel v1 (Integrated DNA Technologies) according to the manufacturer's instructions. All captured libraries were sequenced on an Illumina NovaSeq 6000 system by performing 150 bp paired‐end reads.

The raw data were filtered as follows: (1) remove the adapter sequences from 150 bp paired‐end reads; (2) both reads need to have a quality score (Q‐score) of 30 or higher. Filtered reads mapped to the human reference genome GRCh38/hg38 using the BWA v.0.7.15 MEM algorithm. The Sentieon tool (v2019.11) was used to convert aligned reads to a binary (BAM) file. Genome Analysis Tool Kit (GATK v4) best practices (https://software.broadinstitute.org/gatk/best‐practices/) from the Broad Institute was applied for variant calling (SNP and InDel), and ANNOVAR (http://www.openbioinformatics.org/annovar/) was used for variant annotation including population databases (1000 Genome Project, Exome Variant Server, ExAC, gnomAD, and our in‐house population database), published or submitted variants (HGMD, Clinvar), and in‐silico pathogenicity predictions for missense variants (SIFT, PolyPhen2, LRT, MutationTaster, FATHMM, CADD, REVEL) and splice site variants (MaxEntScan, NNSplice, dbscSNV). Variants were classified according to the guidelines of the American College of Medical Genetics and Genomics (ACMG) (Richards et al., 2015).

Extended WES analysis was done including copy number variant (CNV) analysis by ExomeDepth which used a robust model for the read count data to maximize the power to detect CNV by an optimized reference set (Plagnol et al., 2012) and breakpoint detection to identify structural variations using split‐read, discordant read‐pair, and unmated pairs by SoftSearch (Hart et al., 2013).

## 3 RESULTS

### 3.1 Clinical features

**FIGURE 1:**
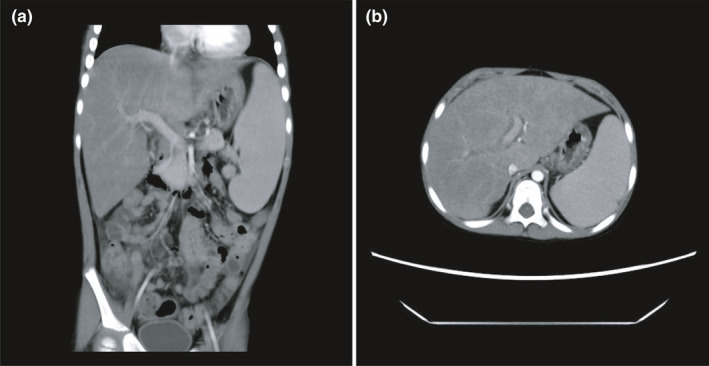
Computed tomography (CT) scan of the upper‐abdomen. The result showed that the patient had both hepatomegaly and splenomegaly. (a) Coronal sections and (b). Axial sections

The patient was a five‐year‐old male of Han Chinese ethnicity (with family from Hunan province, China). He had been well until four years and nine months of age when he was admitted for work‐up with occurred fever, headache, weakness of both lower limbs with pains of unknown origin, and repeated hepatosplenomegaly within 10 months. The results of laboratory testing revealed decreased neutrophils (0.23 × 10^9^/L, 5–12 × 10^9^/L), hemoglobin (73 g/L, 110–160 g/L), C‐reactive protein (13.70 mg/L, 0–8 mg/L), and cytokines (IL‐2, IL‐4, IL‐6, IL‐10, TNF‐α, TNF‐γ) in peripheral blood. Magnetic Resonance Imaging (MRI) results suggested multiple inflammatory lesions in the brain and spinal cord with subarachnoid hemorrhage and cerebral parenchymal hemorrhage. Chest, abdomen, and pelvis CT showed that hepatosplenomegaly and multiple lesions in lungs and kidneys (Figure 1). Cerebrospinal fluid (CSF) examination revealed elevated white cell count (44 × 10^6^/L, 0–20 × 10^6^/L) and protein levels (2.31 g/L, 0–0.5 g/L). The child was diagnosed with septic pyemia initially, with multiple systemic infections. After anti‐infection and symptomatic treatment, the symptoms were partially relieved. Ten months after initial onset, the patient has admitted to the hospital again due to intermittent fever and severe liver dysfunction (alanine aminotransferase 1025.10 IU/L, aspartate aminotransferase 2178.00 IU/L, 0–40.0 IU/L). Further investigation showed increased ferritin (2344.7 ng/ml, 15–152 ng/ml), triglycerides (5.52 mmol/L, 0.40–1.7 mmol/L), decreased fibrinogen (143 mg/dl, 170‐450 mg/dl), and T‐lymphocytes (1368/μl, 1500–2900/μl), B lymphocytes (156/μl, 500–1200/μl) and NK active cells (99/μl, 300–600/μl). Bone marrow cytology revealed changes in hemophagocytosis and blood metagenomic sequencing showed EB virus infection. Immune function assessment displayed increased serum IgG (11.6 g/L, 3.6–10.6 g/L) and IgM (2.42 g/L, 0.38–1.44 g/L) levels. According to the clinical manifestations and diagnostic criteria of HLH, the patient was finally diagnosed with HLH. Finally, the patient was recovered after the hematopoietic stem cell transplantation (HSCT). The preconditioning regimen was VP16+BU+FLU+ATG, with stem cells from Chinese Bone Marrow Bank, HLA 10/10, donor blood type A/RH+, recipient blood type AB/RH+. The patient was followed up 9 months after HSCT, and no post‐transplantation complications occurred.

### 3.2 Identification of genomic structural variants in *SH2D1A* gene

**FIGURE 2:**
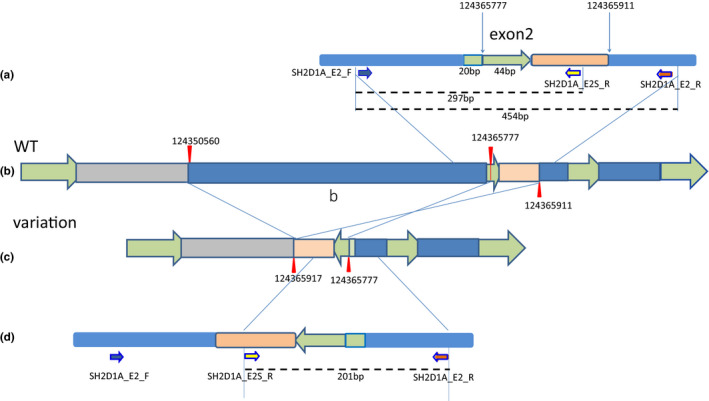
Schematically of SH2D1A genomic structural variation. (a) The exon2 region of SH2D1A. The green arrow showed the exon region. The locis of primer designed in the Inversion‐PCR: SH2D1A_E2_F (blue arrow), SH2D1A_E2_R (brown arrow), SH2D1A_E2S_R (yellow arrow). The DNA fragments in 454 and 297 bp were amplified in wild‐type samples. (b) The genome structure of wild‐type. The red mark labeled 2 breakpoints in intron 2 (chrX:124365911; chrX:124350560), and 1 in exon2 (chrX:124365777). (c) The genome structure is caused by the mutation. (d) 201 bp DNA fragment could be uncovered in the mutation samples

The whole exome sequencing (WES) was performed to further clarify the cause of the disease with recurrent attack and deterioration. However, no candidate variants were identified that could match the patient's clinical phenotype after initially routine analysis. Subsequently, copy number variant (CNV) and structural variations were identified by extended WES analysis to explore any suspicious genomic changes. Breakpoints in exon 2 of *SH2D1A* (chrX:124365777) and intron 2 (chrX:124365911; chrX:124350560) were found (Figure 2b). Subsequently, Inversion‐PCR and Gap‐PCR were designed to further validate the variants in this case.

### 3.3 Verification of structural variation of *SH2D1A* and segregation analysis

Inversion‐PCR was performed to confirm the inversion event. Two pairs of primers (E2_F and E2_R; E2_F and E2S_R) were designed to generate PCR amplifications to cover the breakpoints (Figure 2a,b, Table S1). The 297 and 454 bp DNA fragments should be amplified in the wild‐type (Figure 2a), yet 201 bp DNA fragments amplified in the variant (Figure 2d).

**FIGURE 3:**
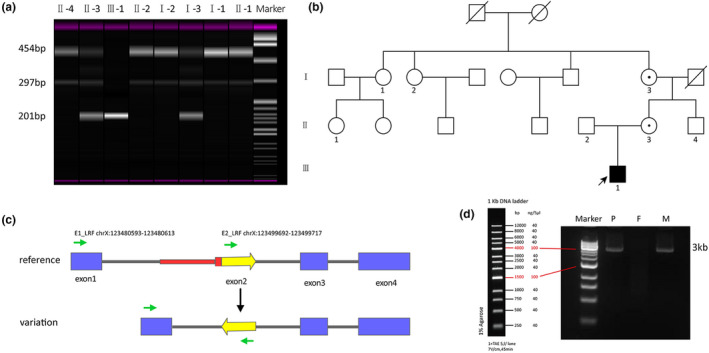
Three‐generation pedigree analysis and Gap‐PCR analysis. (a) Inversion‐PCR results of the amplified fragment in SH2D1A exon 2. The DNA fragments of 201 bp were caused by the identified mutation, and the wild alleles have resulted in both DNA fragments of 454 and 297 bp. (b) Family pedigree of the SH2D1A mutation found in the patient. The white square represents the male members who are normal in this case, the white circle with a dot represents the female members who are a carrier of the X‐linked recessive genetic disorder, the black square with an arrow represents the patient who is the proband. (c) The genome structure and primers (green arrows) of Gap‐PCR. The red region represented the deletion confirmed by Gap‐PCR. The green arrows indicate LRF‐primer and location information were shown in the schematic. (d) The Gap‐PCR products with LRF‐primers were resulting in DNA fragments of 15 kb deletion. M: 1 kb DNA marker; lane P: amplicon of the patient's sample; lane F: amplicon of the father's sample; lane M: amplicon of the mother's sample. Approximately 3.5 kb fragments were amplified from the samples of the patient and his mother

A unique 201 bp capillary electrophoresis PCR product was identified in the patient (hemizygote: III‐1), three mixed PCR products (201, 297, and 454 bp) were observed in I‐3, II‐3 (heterozygote), and two bands (297 and 454 bp) in I‐1, I‐2, II‐1, II‐2, and II‐4 (wild‐type) (Figure 3a). This result confirmed an X‐linked recessive inheritance pattern (Figure 3b) in this family. Gap‐PCR was performed to confirm the large deletion of intron 1 using E1_LRF and E2_LRF (Figure 3c, Table S1). The distance between PCR primers was 18 kb, the wild‐type product was not amplified according to the direction of the primers (Figure 3c). Gel electrophoresis showed 3 kb DNA fragments in the patient and his mother (Figure 3d). This result confirmed a chromosome fragment inversion and 15 kb deletion of *SH2D1A* in exon 2 and intron 1. The location of the breakpoint was obtained by comparison with the second‐generation sequencing reads.

### 3.4 Validation of the mRNA impacts of the genomic structural variants

**FIGURE 4:**
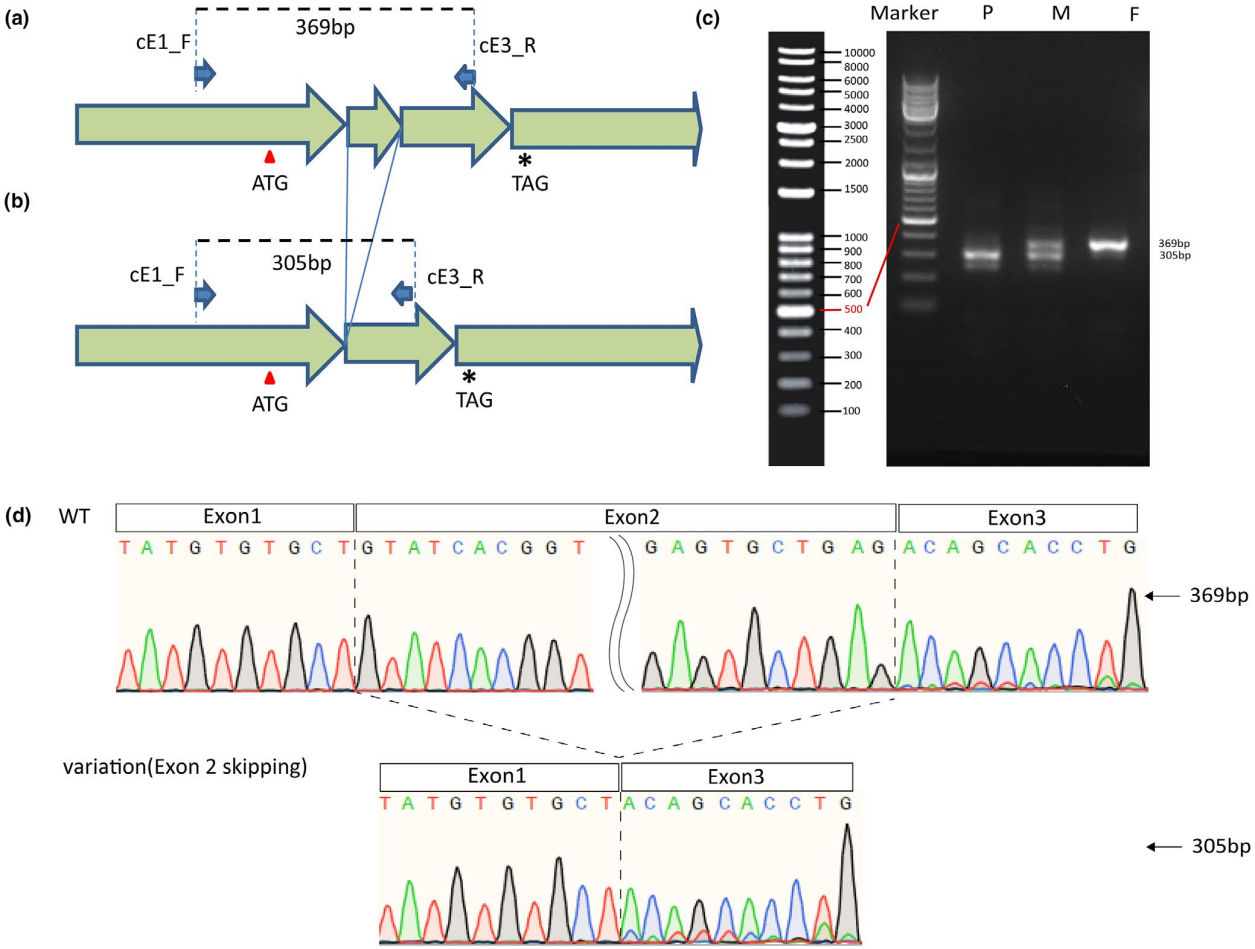
Genomic structural variation caused exon 2 skipping of the transcript. (a,b) Wild‐type and mutant sequence of the transcript. The PCR product of primers named cE1_F and cE1_R would amplify 369 and 305 bp fragments in wild‐type and mutant samples respectively. (c) cDNA amplification result of the proband and his parents. P, M, F showed the PCR products of the proband, mother, and father respectively. (d) The sequencing cDNA amplification product shows that the proband is a direct link between exon1 and exon3, and an exon2 skipping was confirmed

The structural variation involved the splice acceptor site, branch point, and other splicing regulator regions of exon 2 at the genome level. The Reverse Transcription‐Polymerase Chain Reactions (RT‐PCR) were performed to investigate variants functions on mRNA transcription with total RNA extracted from the blood obtained from the patient and his parents. PCR products were 369 bp DNA fragments in wild‐type samples and 305 bp in variant samples using primer cE1_F and cE3_R (Figure 4a,b, Table S1) respectively. Only the truncated DNA fragment of 305 bp was shown due to the variant. Both gel electrophoresis (Figure 4c) and Sanger sequencing (Figure 4d) displayed that the exclusion of exon2 by exon skipping led to the *SH2D1A* mRNA with frame‐shifted exon3 sequences and a premature stop codon (p.Arg47Glyfs*34) of the protein (80 amino acid residues instead of 128 amino acid residues).

## 4 DISCUSSION

We reported a 5‐years‐old male patient, who presented typical features of HLH in clinical examination, including hepatomegaly, low platelet counts, decreased NK cell activity, and hemophagocytosis, and was finally diagnosed with XLP1 based on the molecular testing results. A complex disease‐causing genomic structural variation (NC_000023.11: g. [124,350,560_124365777del; 124,365,777_124365917inv; 124,365,911_124365916del]) was identified by extended WES analysis and amplicon sequencing. In vitro functional validation by gel electrophoresis and Sanger sequencing of the RT‐PCR products showed that the variation causing exon 2 skipping of *SH2D1A* was predicted to produce a truncated protein in the patient. Blood metagenomic sequencing showed EBV infection in the proband pointed toward a failure of the immune system in protecting against EBV invasion, due to elimination of EBV‐infected B lymphocytes by defective Helper T cells (Sumegi et al., 2002).

The *SH2D1A* gene encodes a single SH2 domain protein involved in T‐lymphocytes signal transduction (Morra et al., 2001). Many *SH2D1A* gene variants have been identified in patients with X‐linked lymphoproliferative syndrome. These variants are often associated with either decreased or impaired function of the signaling lymphocytic activation molecule‐associated protein products (Eckrich et al., 2011). The *SH2D1A* cDNA is 2530 bp containing an open reading frame (ORF) of 462 bp and the start codon from ORF is 79 bp. Northern blot analysis with probes generated from the cDNA has shown expression of an approximately 2.5 kb mRNA at a high level in the thymus and lung, but with a lower level in the spleen and liver (Coffey et al., 1998). The considerable dynamics observed in SAP/SH2D1A contribute to its ability to accommodate the non‐optimal sequences. Structural flexibility can improve the stability of various complexes by modulating the binding surface and maintaining the conformational entropy in the absence of favorable interactions. The flexibility of two segments of the BG and EF loops that regulate the peptide binding clef tallows has demonstrated considerable structural plasticity, enabling the extreme versatility of binding specificity observed in this modular domain. Hence, mutations within the boundaries of the SH2 domain could be directly implicated in the pathogenesis of XLP (Hwang et al., 2002; Nichols et al., 1998). However, the genomic structural variation we detected in this study changes the splice site and branch point of exon 2. In consequence, 5’ss and 3’ss are unable to be recognized by the spliceosome complex accurately and exon 2 are skipping finally. cDNA analysis has confirmed the deletion of exon 2 (64 bp). The exon 2 of *SH2D1A* gene encodes 21 amino acids of the SH2 domain that plays a crucial role in binding with SLAM molecule. Thus, dysfunctional SAP protein induces the signal transduction of T‐lymphocytes and displays defects in their regulation. However, the variant spectrum and epidemiological features of this disease in China are still unclear (Jin et al., 2016; Xu et al., 2020). The p.R55X is a hotspot variant in China, which has been identified in 22.9% of Chinese patients with SAP deficiency (Xu et al., 2020). Hence the structure variation of *SH2D1A* gene with HLH is rare in Chinese patients. Our results expand the spectrum of pathogenic variants in *SH2D1A*, and may contribute to further XLP epidemiological surveys.

Initially, we cannot make the molecular diagnosis based on the patient's clinical presentation by routine WES interpretation. More efforts should be done to exploit accurate and robust analysis pipeline and main extended exome variants including copy‐number variation (CNV) (larger CNV and gene or exon level CNV), nonconsensus splice defect detection, genomic breakpoint detection to explore possibilities, which would make whole exome sequencing technology more valuable (Bergant et al., 2018). Indeed, precise XLP1 diagnosis was established through extended WES analysis which significantly modified the therapeutic and follow‐up options for the patients (Vince et al., 2018). Bone marrow or HSCT is currently the only curative treatment for XLP1 patients (Panchal Booth et al., 2018), the survival rate for non‐transplants is below 20% (Booth et al., 2011). Therefore, a clear diagnosis of the patient and the precisive treatment regimen have a positive impact on the prognosis of the disease. For rare disorders, especially due to the high clinical heterogeneity, it is difficult to make a precise diagnosis. WES is an effectively auxiliary diagnostic method to assist the potential diagnostic directions. Now that the patient has recovered from HSCT and this study clarifies the significance of molecular testing in the diagnosis and treatment of rare diseases.

## 5 CONCLUSION

We use extended WES to identify a novel genomic structure variation combined by paracentric inversion and large size deletions of the *SH2D1A* gene in an XLP1 male patient who successfully managed with HSCT. The in vitro studies have shown that the variant functionally disrupted the splice site causing the exon 2 skipping. This finding extends the spectrum of the known XLP‐related mutations in Chinese patients and demonstrates the remarkable effects on the early diagnosis and therapeutic implication if using proper molecular testing techniques.

## CONFLICT OF INTEREST

All authors report no disclosures relevant to the content of this manuscript.

## AUTHOR CONTRIBUTIONS

Conceived and designed the experiments: Jia Wang and Feng Yang. Patient recruitment and clinical analysis: Liwen Wu. WES and molecular analysis: Jia Wang and Feng Yang. Wrote the first draft of the manuscript: Feng Yang, Fan Yang, and Jia Wang. Made critical revisions and approved final version: Liwen Wu, Mengmeng Liang, Feng Yang, Fan Yang, and Jia Wang. The authors reviewed and approved the final manuscript.

## ETHICS APPROVAL AND CONSENT TO PARTICIPATE

Written informed consent was obtained from the patient's legal guardians according to the Declaration of Helsinki. This study was approved by the human ethics committees of The Hunan Children's Hospital.

## Supporting information


Table S1

## Data Availability

The data presented in the study are deposited in the ClinVar (https://www.ncbi.nlm.nih.gov/clinvar/) repository, Accession numbers (VCV001210238). This data is publicly available.
