# Supplementary material for: Exon skipping caused by a complex structural variation in SH2D1A resulted in X‐linked lymphoproliferative syndrome type 1
Source: Mol Genet Genomic Med. 2022 Jan 29;10(3):e1873. doi: 10.1002/mgg3.1873 (PMC8922962; doi:10.1002/mgg3.1873)
Supplement: Supplementary file 1 — Table S1 [file MGG3-10-e1873-s001.docx]

**Supplemental Table 1.** Primers used for the PCRs and sequencing

| *Target region* | *Name* | *Primer sequence (5′–3′)* | *length (bp)* | *temp (°C)* |
| --- | --- | --- | --- | --- |
| Inversion-PCR wild-type I | SH2D1A_E2_F | GGAAACTGTGGTTGGGCAGA | 454 | 60 |
|  | SH2D1A_E2_R | TGCAAAATGATGGCTAAACAGGA |  |  |
| Inversion-PCR wild-type II | SH2D1A_E2_F | GGAAACTGTGGTTGGGCAGA | 297 | 60 |
|  | SH2D1A_E2S_R | ACCTCCTTGACACCCCCAGA |  |  |
| Inversion-PCR mutation | SH2D1A_E2_R | TGCAAAATGATGGCTAAACAGGA | 201 | 60 |
|  | SH2D1A_E2S_R | ACCTCCTTGACACCCCCAGA |  |  |
| Gap-PCR | SH2D1A_E1_LRF | GCGAGAGCGTGCCAGGCGTGT | ~18kb | 68 |
|  | SH2D1A_E2_LRF | TTTGCTTCTGGGGGTGTCAAGGAGGT(ISV) |  |  |
| cDNA exon1-3 | SH2D1A_cE1_F | TCTCCCTTGCACAGTTCTCC | 369 | 60 |
|  | SH2D1A_cE3_R | AGGACTTCTTCTCAACTGGATACTG |  |  |
